# High-Resolution Video Tracking of Locomotion in Adult Drosophila Melanogaster

**DOI:** 10.3791/1096

**Published:** 2009-02-20

**Authors:** Justin B. Slawson, Eugene Z. Kim, Leslie C. Griffith

**Affiliations:** National Center for Behavioral Genomics, Department of Biology, Volen Center for Complex Systems, Brandeis

## Abstract

Flies provide an important model for studying complex behavior due to the plethora of genetic tools available to researchers in this field.  Studying locomotor behavior in *Drosophila melanogaster* relies on the ability to be able to quantify changes in motion during or in response to a given task.  For this reason, a high-resolution video tracking system, such as the one we describe in this paper, is a valuable tool for measuring locomotion in real-time.  Our protocol involves the use of an initial air pulse to break the flies  momentum, followed by a thirty second filming period in a square chamber.  A tracking program is then used to calculate the instantaneous speed of each fly within the chamber in 10 msec increments.  Analysis software then compiles this data, and outputs a variety of parameters such as average speed, max speed, time spent in motion, acceleration, etc.  This protocol will discuss proper feeding and management of flies for behavioral tasks, handling flies without anesthetization or immobilization, setting up a controlled environment, and running the assay from start to finish.

**Figure:**
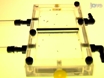


## Protocol

### Part 1: Feeding and Management of flies

1. Flies should be grown in bottles containing yeast-free standard media.  You will need large numbers of flies, so crosses need to be set up accordingly.  Flies should be grown in a 12-hour light:dark cycle at 25ºC.
2. Flies should be collected soon after eclosion (1-3 days).  Flies can be worked with at this stage using a carbon dioxide diffuser, and should be sorted into test tubes containing autoclaved or yeast-free media.  We use males only, stored 10 to a test tube.
3. Allow at least a day or two after use of carbon dioxide before running the behavioral experiments.  We run flies in our behavioral assay 3-5 days following ECLOSION.
4. Assays should always be done within the same 2-3 hour time window each day to avoid circadian rhythm issues.

### Part 2: Setting up the tracking system in a controlled environment

1. All experiments are done in an environment room which maintains a constant temperature of 25ºC with 70% humidity.
2. Suspend a digital video camera over the area to be recorded (camera facing downwards).  Since our tracking software is contrast-based, we suspend the camera over a light box.  The camera records videos directly onto a Dell computer via a firewire connection.  We use a Sharp digital video camera, hooked up to a Dell computer with Windows Moviemaker (Vista version) for video acquisition.
3. Air pulses will be administered in this protocol, so a source for air flow must be present in the room.  Using rubber tubing, connect the air source to a carbon filter for air filtration.
4. Using more rubber tubing, connect the other side of the carbon filter to an Erlenmeyer flask via a hollow rubber stopper, and fill the flask with about a half inch of water.  This will humidify the air.
5. The flask should also have a sidearm, which will connect to a Y-shaped valve via rubber tubing.
6. One branch of the Y-valve should be connected to a flowmeter.  The other branch should deliver air to the square locomotion chamber.

### Part 3: Delivering flies to the locomotion chamber

1. The square locomotion chamber is based on the design from Wolf *et al.* 2002^1^, with the addition of small drop trays to prevent the flies from flying within the chamber.  The chamber should be taken apart, and all components should be thoroughly wiped down with 70% ethanol and dried before use.
2. Anesthesia immediately before conducting a behavioral assay can potentially compromise performance. To avoid this, flies are gently knocked into the chamber using a funnel with a small outlet.  To make an outlet of the right size, attach a blue pipette tip (for P1000) to the end of a funnel.  Use scissors to cut off the very end of the pipette tip to make the opening big enough for a fly to pass through.
3. The top of our chamber is a small piece of plexiglass secured by screws around the edges.  When the screws are removed, the holes can be used to deliver flies into the chamber.  Position the plexiglass top so that the screw hole sits directly over the inner chamber instead of where a screw normally sits.  Take a test tube containing the flies and place it upside down over the funnel, which should then be placed into the screw hole.
4. Do NOT move the funnel, as this could hurt or damage the flies.  Instead, gently bang the whole chamber, funnel and all, until all flies are inside the chamber.  This knocking should be done on a mouse pad to absorb the shock of this banging. When flies are inside, remove the funnel, and re-position the plexiglass top over the screw beds.  Then re-screw the plexiglass top into place.

### Part 4: Running the Locomotor Assay

1. Once the flies are properly loaded, let them acclimate in the chamber for 30 minutes.  This acclimation period should take place in the controlled environment (25ºC, 70% humidity), and the chamber should be placed on top of the light box (which should be turned on).  Turn other room lights off.
2. Following the acclimation period, switch the Y-valve in the air flow system such that the air will only flow to the flowmeter.  Turn on the air, and ramp the air up to the desired speed (4.0-6.0 L/min).  We generally use 5.0-5.5 L/min, but any speed in that range will work.
3. Once the desired airspeed is attained, switch the Y-valve so that the air flows to the locomotion chamber.  Time this air pulse for 15 seconds, and then abruptly turn the air off. As you turn the air off, switch the camera to record mode. (This part takes practice to do all at once).
4. Stop the recording after the desired time and SAVE.  We record 30 second trials at 10 frames per second (at least 8 trials per genotype).

### Part 5: Analysis of Videos

1. We use the Dynamic Image Analysis System (DIAS) software 3.2 for motion tracking^2^.  In order to use this software, we first convert our videos to an AVI file format with Quicktime 7.5.5.
2. To track the flies based on contrast, we use the “Autotrace by Threshold” function. We then use the “Make Path from Trace” function to digitize these traces.
3. To output the instantaneous speeds of each fly, we use the “Compute Parameters” function to produce a database file (DIAS-specific).  Using this function, we also smooth the data using a “5,15,60,15,5” Tukey Smoothing Window.
4. Once this information is outputted as a database file, it can be opened in Microsoft Excel. We use a Matlab script to compile the instantaneous speeds within excel and calculate additional parameters necessary for understanding movement dynamics and bout structure.

### Representative Results:

Figure 1 shows representative traces from wild type *Canton S *organisms (Figure 1, left panel) and flies null for the *dCASK* gene which were generated by crossing two large overlapping deletions (*Df(3R)x307 *and *Df(3R)x313*) (Figure 1, right panel).  *dCASK* null flies have previously been shown to have locomotor problems using Buridan’s Paradigm^3^, and in our paradigm, compared to Canton S, they show greatly decreased locomotion.  We always run wild type organisms first to make sure that conditions and behavior are within the normal range on a given day.  We have observed deviations from standard responses in less than 5% of testing days.  These differences can usually be attributed to problems with parameters of our controlled environment.


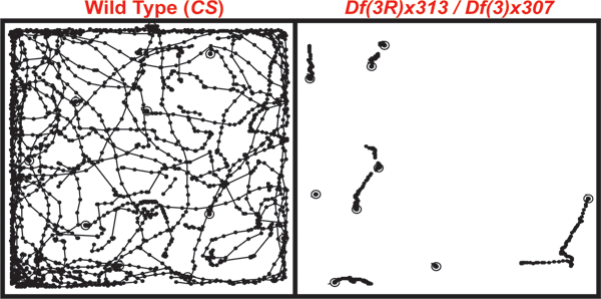


**Figure 1.** DIAS-generated traces of both *Canton S* wild type flies (above, left) and Locomotor-deficient *dCASK* Null flies generated from overlapping deletions (above,right). The traces represent what is seen in the recorded videos. Both pictures contrain traces from 8-10 flies run for 30 seconds in the video tracking assay following an air pulse.


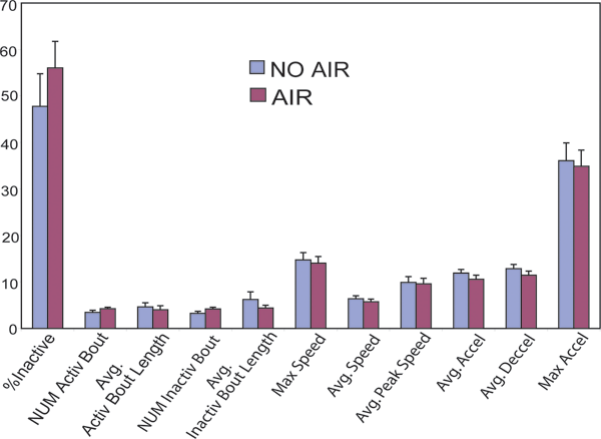


**Figure 2.** Wild type flies were run in the locomotor assay (above) following an air pulse (purple bars) and without an air pulse (blue). In all parameters calculated by the analysis program, there were no significant differences between the two conditions (determined with two-tailed student t-test). This demonstrates that following an air pulse, flies locomote normally in our setup.

## Discussion

In our setup, a moderately strong air pulse transiently stops fly movement.  When the air pulse ends, the flies are released from this stationary state and locomote normally, as shown in figure 2.  Because this air pulse effectively synchronizes locomotion of the population, we use it to begin the trial so that we can also study the onset of movement following a momentum-breaking stimulus.  The assay can, however, be done without an air pulse since locomotor parameters following initiation are unaffected by the air pulse (Figure 2).

One frequently occurring problem seen with automated analysis of locomotion is the issue of collisions.  Tracking programs are for the most part notoriously bad at dealing with flies that collide. These programs often lose “sight” of an object momentarily during a collision, and upon finding this object label it as a new object.  The result can be an output that has many more objects traced than actually exist in the chamber.  Many people solve this problem by tracing single flies.  The obvious problem with this, however, is that single fly behavior may be very different than population behavior due to the role of social cues in *Drosophila* behavior and locomotion^4^.  This is, of course, not necessarily a bad thing, depending on what you are trying to study, but for our purposes, we prefer to use populations of flies to increase our statistical power.  Because of this, we deal with collisions in two ways.  First, we only use 8-10 flies per trial (in our 56 mm sq chamber), so that collisions will be minimal.  Second, within our thirty second trials, we only analyze traces that are at least 18 seconds in length.  By doing this, we are always recording enough time to capture multiple bouts of motion in their entirety.  This also ensures that our sample size always reflects the number of flies in a chamber, as smaller fragments of motion are discarded and the total length of the video is only 30 seconds.

Data analysis can be the most difficult part of this whole process.  The most important step of analyzing tracking data is to determine the difference between noise and motion.  DIAS (like many other programs) will almost never output a speed of 0 mm/s, even if the fly has stopped moving.  Because of this, it is important to watch the videos while looking at the data output frame by frame to see when the organisms are actually in motion, and when they are not.  In our setup, all speeds below 1 mm/s seem to be noise, which is consistent with what other groups using DIAS have found with adult flies^1^.  For looking at the dynamics of bout structure, one also needs to define a bout.  We define activity as 3 or more consecutive frames of speed above 1 mm/s, and inactivity as 3 or more consecutive frames below this threshold.

The data must also be properly smoothed to eliminate artifacts from transient light flickering and camera distortions, which occasionally occur.  There is no standard method for data smoothing, but it is important that the smoothing process does not change the general trend of the data too drastically, or one can generate smoothing artifacts.  We smooth twice with a Tukey window of “5,15,60,15,5”, because it seems to eliminate any large jumps or changes in speed that are clearly wrong, but does not change the overall trend or nature of the data.

It is important to recognize that different setups and environments will produce different behavioral outcomes.  Our recommendation to anyone setting up a tracking assay is to experiment with all of these issues until you find a method which produces data that matches what can be seen visually, and then be as consistent as possible in treating all data in the same manner.

Selection of the right tracking program is also important.  Although we use DIAS 3.2 (www.solltechnologies.com), this is by no means the best or the only system available.  For tracking of single flies, Dan Valente (Mitra Lab, CSHL) has developed a program called Ftrack.  For multiple flies locomoting together (i.e., where collisions might occur), Kristin Branson (Dickinson/Perona Labs, Caltech) has developed a program called Ctrax.  Ftrack is available at www.chronux.org, while Ctrax is available at www.dickinson.caltech.edu/Research/Mtrax.  Ethovision software (Noldus, Netherlands) is another powerful option available for video tracking of both single and multiple flies, but it is only commercially available, and is quite expensive.

If you have set up a tracking assay and cannot get reliable recordings, there are a few things to consider.  Circadian rhythm issues are often a big problem.  When running behavioral assays, one should always make sure NOT to run flies during their midday siesta.  Since we are currently interested in genes that can produce decreases in locomotor behavior, we prefer to run flies near the late afternoon activity peak (ZT 8-10).  Another problem can also arise from inconsistent air flow from the source.  Before starting trials, be sure to test the air flow with the flowmeter, so that it will not fluctuate greatly over the 15 second air pulse.  Lastly, genetic background can play a role in all behavioral tasks, so some of the parameters of this assay may need to be optimized for a particular background.
